# A New and Worrisome Predictor of Attitudes Toward Suicide: Self-Rated Health

**DOI:** 10.1093/geroni/igab046.1469

**Published:** 2021-12-17

**Authors:** Julene Cooney

**Affiliations:** Syracuse University, Manlius, New York, United States

## Abstract

Do people have the right to end their own lives? The General Social Survey has monitored the attitudes of Americans towards suicide since 1977 using four questions: Do you think a person has the right to end his or her own life if this person has an incurable disease, has gone bankrupt, has dishonored his or her family, or is tired of living and ready to die? These four responses can be combined into a reliable index representing an individual’s attitude toward suicide. As average population education levels have increased and religiosity has fallen, attitudes favoring the right to suicide have increased across the population. This research project introduces a previously understudied predictor of attitudes toward suicide: self-rated health. Using logistic and ordinal logistic regression, and controlling for age, education level, religiosity, marital status, survey year, race, and sex, I find that, over time, self-rated health has become a significant predictor of attitudes toward suicide. Since 2002, respondents who perceived themselves to be in poor health are significantly more likely to favor the right to end one’s life, especially if the individual has an incurable disease. After stratifying by age and race, I find that the relationship between self-rated health and attitudes toward suicide is strongest among individuals in the mid-life and is equally significant as a predictor for White and Black Americans after 2010. These findings provide further evidence that mental health screening is an increasingly vital component of physician/patient interactions and highlight the importance of continuity of care.

